# Robust Iris Segmentation Algorithm in Non-Cooperative Environments Using Interleaved Residual U-Net

**DOI:** 10.3390/s21041434

**Published:** 2021-02-18

**Authors:** Yung-Hui Li, Wenny Ramadha Putri, Muhammad Saqlain Aslam, Ching-Chun Chang

**Affiliations:** 1 Department of Computer Science and Information Engineering, National Central University, Taoyuan 32001, Taiwan; yunghui@csie.ncu.edu.tw (Y.-H.L.); wenny@g.ncu.edu.tw (W.R.P.); saqlain@g.ncu.edu.tw (M.S.A.); 2 Department of Electronic Engineering, Tsinghua University, Beijing 100084, China

**Keywords:** iris recognition, iris segmentation, deep convolution and deconvolution neural network, image segmentation, biometrics

## Abstract

Iris segmentation plays an important and significant role in the iris recognition system. The prerequisite for accurate iris recognition is the correctness of iris segmentation. However, the efficiency and robustness of traditional iris segmentation methods are severely challenged in a non-cooperative environment because of unfavorable factors, for instance, occlusion, blur, low resolution, off-axis, motion, and specular reflections. All of the above factors seriously reduce the accuracy of iris segmentation. In this paper, we present a novel iris segmentation algorithm that localizes the outer and inner boundaries of the iris image. We propose a neural network model called “Interleaved Residual U-Net” (IRUNet) for semantic segmentation and iris mask synthesis. The K-means clustering is applied to select saliency points set in order to recover the outer boundary of the iris, whereas the inner border is recovered by selecting another set of saliency points on the inner side of the mask. Experimental results demonstrate that the proposed iris segmentation algorithm can achieve the mean IOU value of 98.9% and 97.7% for inner and outer boundary estimation, respectively, which outperforms the existing approaches on the challenging CASIA-Iris-Thousand database.

## 1. Introduction

Recent developments in the field of computer vision have led to renewed interest in biometrics technologies. Iris recognition has been determined as the most accurate and reliable biometric identification approach and thus it has been deployed in several applications such as identification and authentication systems, intelligent key systems, digital forensics, and border control [1,2,3,4]. An iris comprises a large amount of distinctive, constant, and forgery-proof features such as complex textures and explicit structural information for biometric identification [5,6]. In addition to this, iris traits are stable and remain unchanged throughout a person’s lifetime [7]. Hence, iris texture serves a key role in biometric practices.

**Figure 1:**
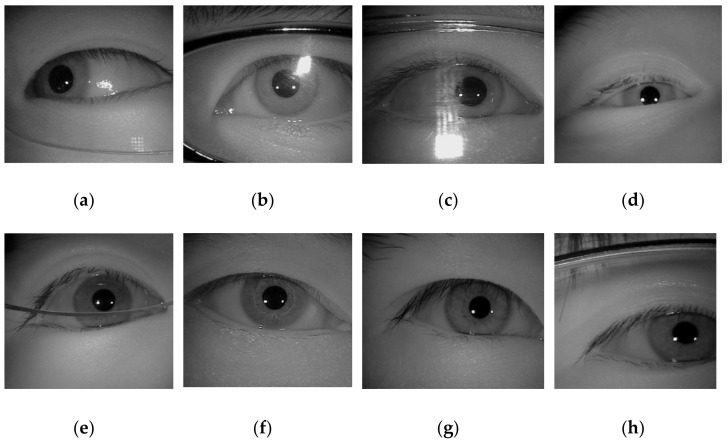
Example of images captured under non-cooperative environments. (**a**) Off-angle iris; (**b**–**c**) iris with specular reflection; (**d**) iris occluded by eyelids; (**e**) iris obstruction due to glasses; (**f**) rotated iris image; (**g**) iris obstruction due to eyelash; (**h**) partially captured iris.

Over the last few years, with the rapid advancement of technology, the use of mobile devices like smartphones, computers, or smartwatches have exponentially increased in our daily life. Meanwhile, the amount of user’s secret data stored in mobile devices is constantly growing. Hence, biometric verification is needed to restrain unknown users from gaining access and stealing personal sensitive information stored on mobile and handheld devices. Compared with lock patterns, PIN codes, and passwords, iris patterns not only provide stronger protection for mobile devices against digital threats, but are also more reliable, anti-counterfeit, comfortable, and user-friendly than entering a pin code or password to unlock the device [1,8]. In recent years, fingerprint scanning could be recognized as the most ubiquitous biometric function embedded in mobile and handheld devices [9]. However, there have been spoofing cases, such as fingerprints picked up from the touchscreen. Comparatively, the iris-based system is foolproof and quite convenient to implement although it may require an infrared sensor and specially designed optics [1,9]. However, iris segmentation tasks are challenging because the acquisition of iris images may suffer from non-cooperative environments including specular reflection, off-angle iris, occlusion due to eyelash/eyelid/glasses, partially captured iris, etc., as shown in Figure 1 [3].

A common iris recognition system often consists of the following procedures: Iris image acquisition, iris image pre-processing, iris segmentation, iris feature extraction, and feature matching for identification or authentication [1,3,4,6,10]. In the iris recognition system, iris segmentation is a critical and challenging task because of the unpredictable and irregular shape of the iris [4,7]. In general, iris textures are more visible near the area close to the pupil boundary. If the boundary of the pupil area is not correctly positioned, a large amount of iris texture will be lost in the feature extraction stage. In other words, the performance and robustness of the iris recognition system depend strongly on the accuracy of iris segmentation. In most cases, the occluded iris area (e.g., eyelids or eyelashes, eyeglasses, poor illuminations, and motion blurs) would greatly influence the overall accuracy of iris segmentation algorithms. Previous studies have shown that the error produced in the iris segmentation step is transmitted into all following phases of the iris recognition, so iris boundary estimation is still a very important pre-processing stage for achieving the high accuracy of the system [1,2,4,6].

In this paper, we attempt to address this problem. To this end, we propose a multi-stage algorithm, which consists of deep learning and saliency points selection for iris. Our deep learning model is based on an interleaved residual U-Net (IRUNet), which is more effective and robust in terms of locating the iris area accurately. The proposed method splits the task of iris segmentation into two processes: (1) Finding the eye and (2) segmenting the iris area. Our well-developed IRUNet network model is used to generate an iris mask which is capable of estimating the boundary points correctly, whereas a K-means clustering is used to improve the iris boundary points from non-saliency points.

The rest of the paper is organized into the following sections. Section 2 describes the related work. In Section 3, the proposed method is presented. Experimental results and discussion is presented in Section 4, and Section 5 draws the conclusion.

## 2. Related Works

The recent algorithms for iris segmentation can be classified into the following kinds depending on the implementation methods. Conventional iris segmentation approaches are based on a boundary-based schema.

In these methods, the inner and outer boundaries of the iris are initially identified, and then filtered iris masks are acquired through detecting the lower and upper eyelids and limbic regions. This means that iris localization is performed first, and then the iris segmentation process is performed [1,2,3]. The two traditional and most popular algorithms for iris segmentation were introduced by Professor Daugman’s integrodifferential operator [11] and Wilde’s circular Hough transforms [12]. These algorithms rely on the following idea: Find edge points in the iris image, and then use a circular or elliptical model for fitting. Based on these benchmark algorithms, a large number of later introduced methods achieved significant enhancements in terms of proficiency, robustness, and accuracy [3], for instance, [13] implemented a region-based clustering namely fuzzy-clustering algorithm, heretofore localization to decrease the parameter search range. Tan et al. [14] proposed a combination approach of region clustering i.e., eight-neighbor connection clustering, to cluster the entire iris image into different segments, semantic refinements to extract actual iris area, and then integrodifferential constellation is applied to decrease the reckoning time of the integrodifferential operator. Betancourt and Silvente [15] presented an iris localization approach based on the analysis of gradient approximation. The aggregation operators, quantified majority operator (QMA-OWA) [16], were used to attain iris circular boundaries. Ghodrati et al. [17] proposed a localization algorithm and applied a set of morphological operators, canny edge detector [18], and Hough transforms. Wang and Xiao [19] proposed an iris segmentation approach that relies on the difference operator of radial directions.

Some of the researchers applied region growing algorithms rather than edge-based methods. These kinds of algorithms are related to those for identifying the real iris boundary. They slowly joined the sections with high similarity in an image to acquire the iris area. Liu et al. [20] presented a novel iris segmentation algorithm based on K-means clustering. The proposed limbic boundary localization method utilized a K-means cluster to detect the pupillary region. Yan et al. [21] used the watershed transform [22] and region merging on the structured eye images. Abate et al. [23] combined the watershed transform, color quantization, and region merging.

Furthermore, to acquire the iris mask in the above-mentioned auxiliary methods, there are some pixel-based iris segmentation methods to detect the iris and non-iris areas directly. They generally utilize the low-level visual information (such as intensity and color) of each pixel to categorize the pixel of interest from the image background [3]. Principally, favorable approaches generally practice well-known pixel-level methods, for example, GrowCut [24], geodesic active contours (GAC), and GrabCut [25], to preprocess the image and next implement conventional classification techniques, e.g., Support Vector Machine (SVM) [26], to categorize iris and non-iris pixels.

Recently, in the field of computer vision, deep learning (DL) has gained considerable research attention. Among all DL models, convolutional neural networks (CNNs) have achieved state-of-the-art performance and accuracy in object detection and classification tasks. The two traditional CNN-based models for iris segmentation were introduced by Liu et al. [27], i.e., hierarchical convolutional neural networks (HCNNs) and multi-stage fully convolutional networks (MFCNs). Both of these models find iris pixels automatically without manually designed (handcrafted) rules or features. They are end-to-end prediction models where classifiers and features are jointly optimized and need no more pre- and post-processing. Thereafter, scholars have implemented pre-designed (off-the-shelf, existing) [28,29,30,31] or custom-built [1,32,33] fully connected networks (FCN) models for iris segmentation and achieved good segmentation accuracy on various iris databases. In Lian et al. [31], Lozej et al. [34], Wu and Zhao [35], and Zhang et al. [36], scholars employed alternatives of U-Net [37] for iris segmentation. Despite the success of U-Net, these schemes still have some shortcomings [38], as described below: (1) The skip connection unreasonably forces the aggregation of the same scale feature maps of the encoder and decoder; (2) the optimal depth of the model is not yet known, so immense architecture searches are required, which sometimes leads to invalid collections of models with different depths. The authors in Wang et al. [39] proposed a method for accurate scleral segmentation by advancing a U-Net model called ScleraSegNet. They performed a comparison of various attention modules and obtained a high-performance by using channel-based attention.

To overcome the weaknesses of previous approaches, a robust, effective, and well-designed iris segmentation method is desirable in the research community. In this research, we propose a novel iris segmentation method based on an interleaved residual U-Net (IRUNet). The main contributions of this paper are (1) a novel architecture utilizing interleaved residual U-net for highly accurate iris mask estimation; (2) a novel method called Scan-and-Cluster Strategy, which is able to locate the inner and outer boundary given an iris mask. The detail of the proposed method is illustrated in Section 3.

## 3. Proposed Method

The proposed method consists of three steps: Iris mask estimation, outer boundary estimation, and inner boundary estimation. For the iris segmentation step, we designed an IRUNet model to do semantic segmentation and generate masks. Then, the outer boundary was found by using K-means clustering for the saliency points and the inner boundary was found by saliency points selection from the outer boundary.

### 3.1. Iris Mask Estimation

**Figure 2:**
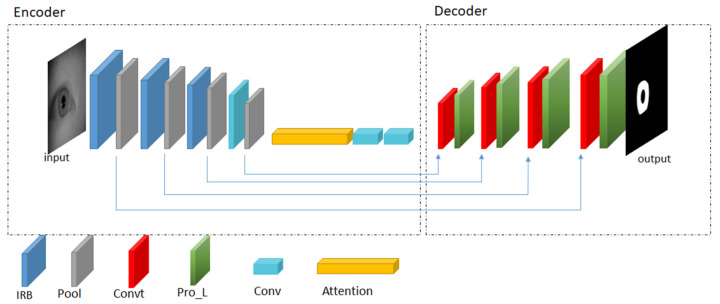
The architecture of self-designed IRUNet. The abbreviation ‘IRB’, ‘Pool’, ‘Convt’, ‘Pro_L’, ‘Conv’, and Attention stands for interleaved residual block layers, pooling layers, transpose convolutional layers, process layers, convolutional layers, and attention module, respectively.

The final goal of iris segmentation is to estimate the location of the inner and the outer boundaries. In this work, we assume that the inner and the outer boundary can be approximated using the circular parameter. However, it can be easily adapted to ellipse by fitting the recovered saliency points with the elliptical equation. There are many ways to achieve such a goal. One of the best practices is to estimate the iris region and derive an accurate iris mask. An iris mask is a binary image of the same size of the input image, with pixel value zero indicating all locations, which does not belong to iris textures. In this work, we designed an IRUNet based on U-Net [37] to achieve this goal. Compared to the original U-Net, the self-designed IRUNet consists of two parts: Encoder and decoder. In the encoder part, it performs downsampling operations and extracts useful features from images. The decoder part consists of multiple upsampling operations and transpose convolution layers. The IRUNet model changes the structures of conventional CNNs, which only consist of an encoder layer. The network architecture is shown in Figure 2.

**Figure 3:**
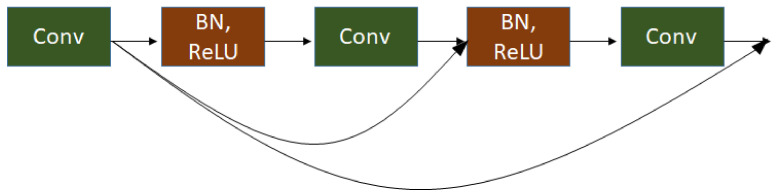
Interleaved residual block with 3 convolutional layers.

As shown in Figure 2, the left part of IRUNet is the encoder part and the right part of IRUNet is the decoder part. First, the convolutional layer 1 × 1 is used to convolve the input images which has 64 kernels with a 3 × 3 size and stride 1. The network has three IR blocks followed by pooling layers and an Atrous Spatial Pyramid Pooling (ASPP) inspired by Deeplabv3 [40] to extract multi-scale features yet keeping the resolution unchanged. The interleaved residual block is adopted in this architecture to prevent the gradient vanishing and overfitting effectively due to the limitation of deep CNNs. This block consists of three convolutional layers, each followed by batch normalization (BN) layers and rectified linear unit (ReLU) activation layers. The connection is applied to connect the first convolutional layers to the later convolutional layers in order to integrate the small feature value with the larger feature value. The detail illustration of interleaved residual block is shown in Figure 3.

**Figure 4:**
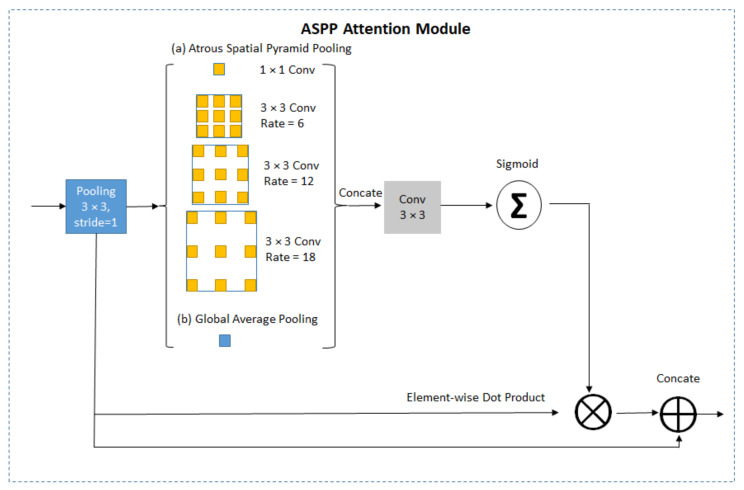
Illustration of Atrous Spatial Pyramid Pooling (ASPP) attention module.

The attention module implemented a dilated convolution with dilation rate equal to 6, 12, and 18 for every convolution layer. The details of ASPP attention module is illustrated in Figure 4. Transpose convolutional layers adopted on the decoder part perform upsampling operations followed by process layers to fuse the output of transpose convolution with pooling layers from the encoder part. The prediction layers obtained prediction masks with the pixel values zero or one, where zero denotes the non-iris pixels and one denotes the iris pixels. The padding is zero for all convolutional kernels.

### 3.2. Scan-and-Cluster Strategy for Saliency Points Estimation for Outer Boundary

After we obtain the iris mask from the IRUNet model, we need to estimate saliency points on the outer boundary of the iris. There are many points that can be extracted from the edge map of the estimated iris mask. However, not all of the points are usable for fitting the elliptical of the boundary. For example, in the non-cooperative environment, the user may wear eye glasses when the iris image is captured. The specular reflection caused by the glass lens may occupy partial iris region. Therefore, parts of the edge that appears on the estimated iris mask may come from the specular reflection (or upper and lower eyelid), instead of authentic iris boundary. That is why it is important to select saliency points from the edge map and ignore the non-saliency points. In this work, we apply a strategy called “Scan-and-Cluster” to discover the saliency points of the iris outer boundary. First, we search for the iris region in order to determine the scanning points area. Because the upper and lower eyelid might occlude the upper and lower part of the iris, the scanning area is defined to only include the range from 0.25× to 0.75× height of the region of interest (ROI) for the iris region. Second, we search from all the points within the range located at the right and left side of iris mask. Third, we form pairs of points by grouping two points, one from the left side and the other from the right side. The distance between the paired points are used to provide robust estimations of the diameter for the circle of the outer boundary. Therefore, the principle for pair selection is to select the paired points that have the longest distance with each other. The procedure of “scan” starts at making the first line segment connecting the uppermost point of the left edge with the lowermost point of the right edge. Then, the second line segment is made by connecting the second uppermost point of the left edge with the second lowermost point of the right edge. This procedure continues in such fashion until all possible pairs of points between the left and right edge are grouped with lines. Assuming $\vec{u}\left(u_{1},u_{2}\right)$ and $\vec{v}\left(v_{1},v_{2}\right)$ denote coordinates of the two points on the left edge and right edge of a line segment, the Euclidean distance $d\left(\vec{u},\vec{v}\right)$ between them can be computed using Equation (1):

(1) $$d\left(\vec{u},\vec{v}\right)=\sqrt{{\left(u_{1}-v_{1}\right)}^{2}+{\left(u_{2}-v_{2}\right)}^{2}}$$

**Figure 5:**
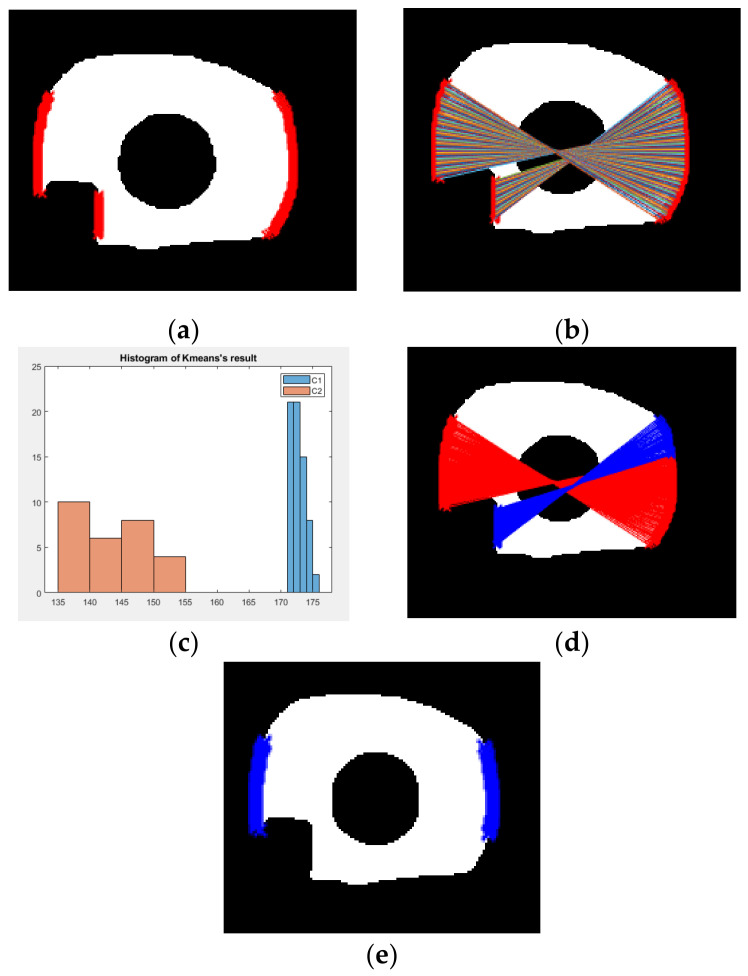
The illustration of the algorithm “Scan-and-Cluster” to find the saliency points of outer boundary. (**a**) Red dots denote all boundary points on the left and right side of region of interest (ROI) (within the range between 0.25× and 0.75× height of iris mask region); (**b**) paired iris points used to compute the distance; (**c**) histogram distribution of the length of lines; (**d**) the paired points of two cluster (red lines denote cluster one and blue lines denote cluster two); (**e**) blue dots denote all recovered saliency points after applying Scan-and-Cluster.

**Figure 6:**
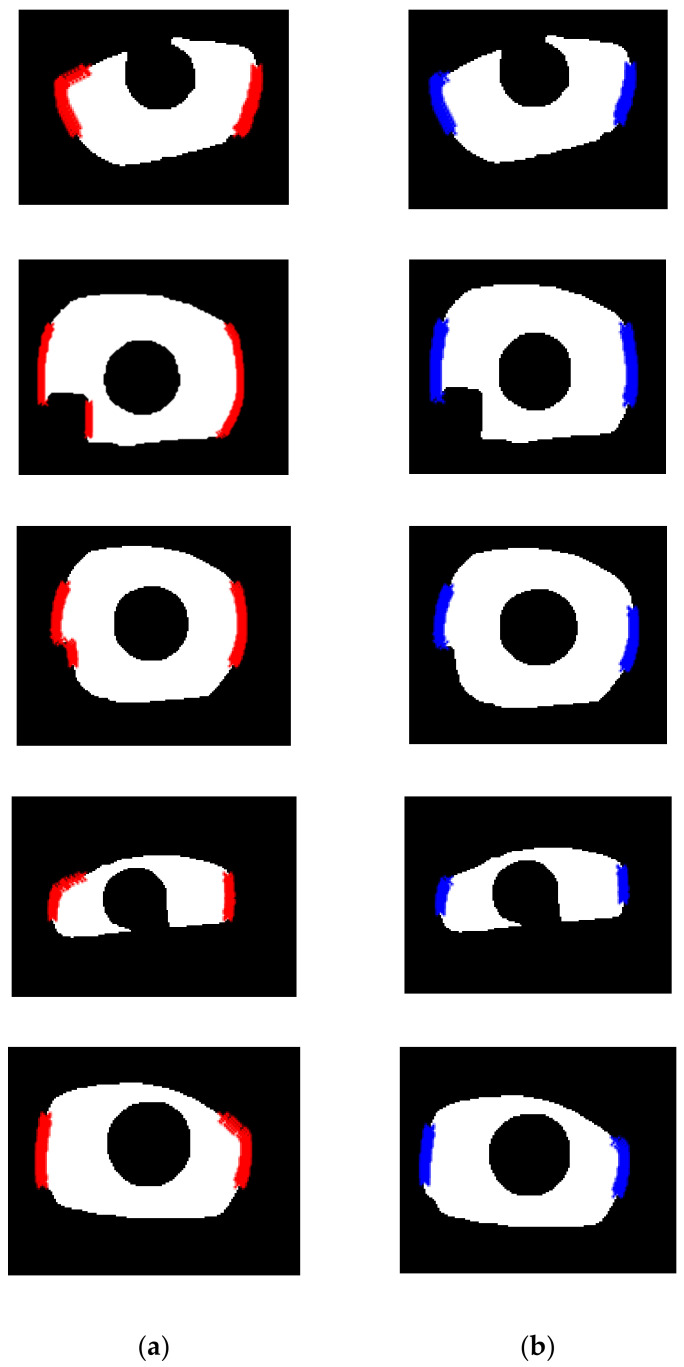
Examples of saliency points estimation result before and after applying “Scan-and-Cluster”. (**a**) Red dots denote all candidate points before Scan-and-Cluster; (**b**) blue dots denote recovered saliency points after Scan-and-Cluster.

This procedure is applied to all line segments. Figure 5a shows an example of the candidate points on the left and right side of an exemplar iris mask. Figure 5b shows all the line segments connecting the possible pairs in the procedure of scan. After we compute the distance for all possible pairs, we perform K-means clustering [41] with the distance values. Figure 5c shows the histogram distribution of the distance value computed from Figure 5b. It can be observed that there indeed exist two distributions. The group of line segments corresponding to the higher and lower values are drawn with red color and blue color, respectively, in Figure 5d. In Figure 5d, we can see that the end points of the red line segments are points located on the iris boundary, but for the end points of blue line segments, some of them are located on the edge of specular reflection. Therefore, the end point of the red line segments are the saliency points that we look for. Figure 5e shows the recovered saliency point with blue color. More examples before and after the algorithm “Scan-and-Cluster” are shown in Figure 6. The detailed procedure for “Scan-and-Cluster” is described in Algorithm 1. After the saliency points are found, to recover the parameter of the outer boundary circle, we solve the optimization problem defined in Equation (2):

(2) $$\left(x,y,r\right)=\arg min_{x,y,r}\overset{N}{\underset{i=1}{\sum }}{\left(x_{i}-x\right)}^{2}+{\left(y_{i}-y\right)}^{2}-r^{2},$$

where $\left(x_{i},\;y_{i}\right)$ denotes the coordinates of the $i^{th}$ recovered saliency point, and *N* denotes the number of all recovered saliency points.

**Algorithm 1.** The algorithm of Scan-and-Cluster for saliency points estimation of iris outer boundary.(1) Define the scanning area from 0.25× to 0.75× height of the ROI of estimated iris mask.(2) Search all the points within the range for the right and left side of ROI.(3) Repeatedly do following step for all points:  (a) Compute all possible paired distance of two points using Equation (1).  (b) Find the maximum value of distance.  (c) Form a set Ω consisting of all distance values computed from (b).(4) Apply K-means algorithm with the value of k = 2 on set Ω. Suppose the subset Θ denotes the cluster that has higher value.(5) Saliency points are recovered as all the end points corresponding to all lines that belonged to Θ.

### 3.3. Saliency Points Estimation for Inner Boundary

**Figure 7:**
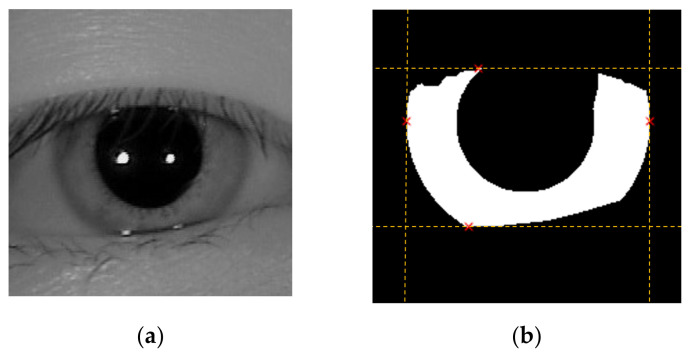

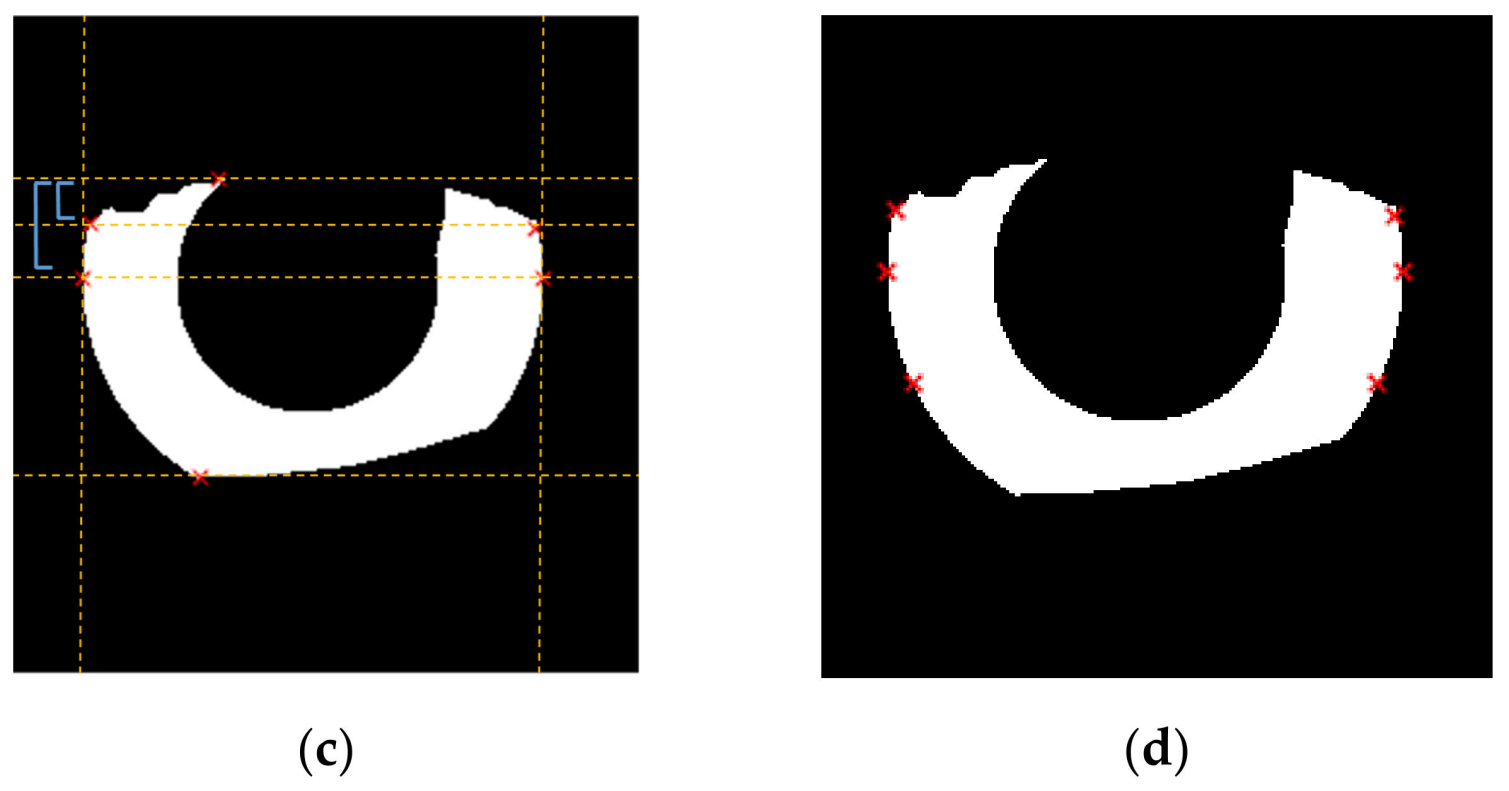
A pictorial example to show how to recover six saliency points for estimating inner boundary. (**a**) The iris image whose upper part is occluded by the eyelid; (**b**) the iris mask ROI (derived from the proposed IRUNet) and the left-most, right-most, top-most, and bottom-most endpoints of ROI; (**c**) the two saliency points on the upper part of ROI is derived by finding the intersection between the ROI and the scan line, which has equal distance to the top-most and left-most endpoint; (**d**) the six saliency points recovered by the proposed method.

The procedure for selecting the saliency points for inner boundary was initialized by finding the left-most, right-most, top-most, and bottom-most endpoints of ROI. It is likely that eyelids might obscure the iris image around the upper and lower region of the pupil area, hence we ignore the top-most and bottom-most endpoint and replace them with other candidates. To search for good saliency candidates, we insert a new scan line in the middle between the upper-most endpoint and horizontal line that passes through the left-most endpoints. The recovered saliency point is the point where the scan line intersects with the iris mask. This procedure is implemented to both the left and right parts of the iris mask to discover two saliency points, one for each side. The same procedure is repeated for the bottom-most endpoint. In the end, we recover six saliency points and ignore the top-most and bottom-most points. A pictorial example of this algorithm is shown in Figure 7.

**Figure 8:**
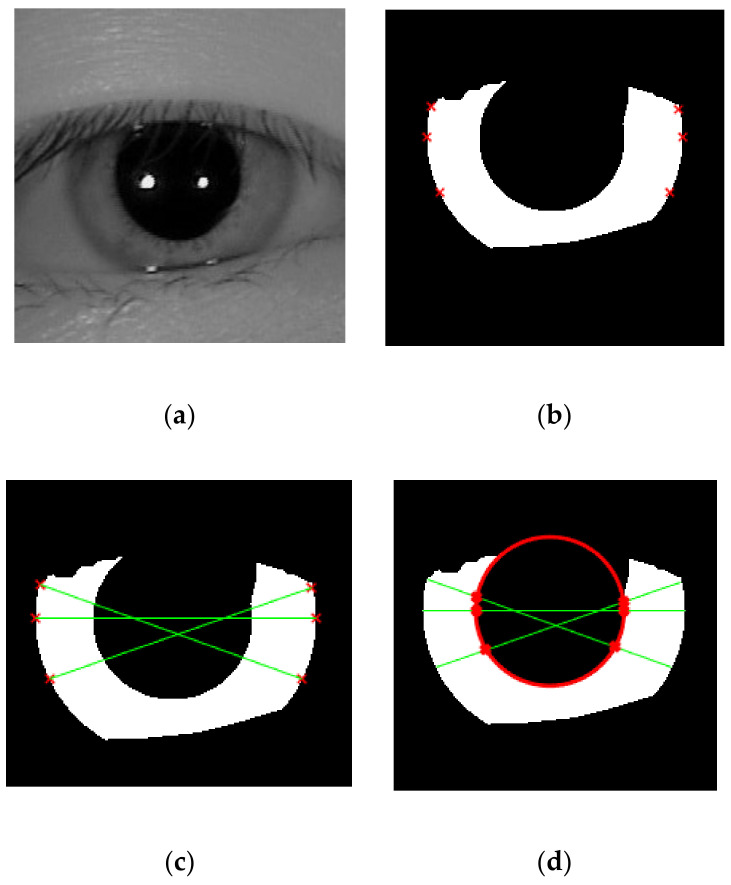

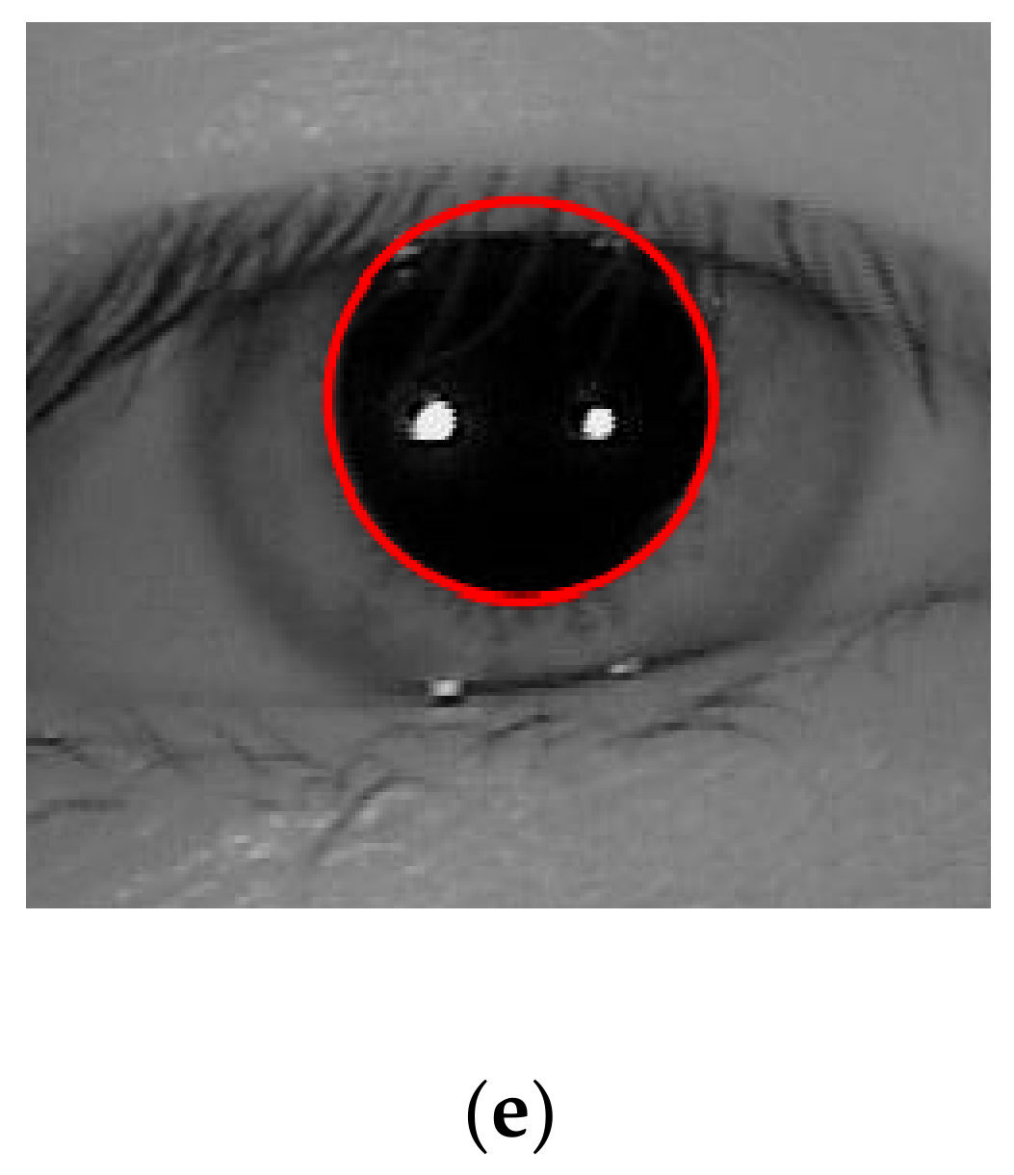
A pictorial example to show how to recover the inner boundary from the six saliency points derived from Figure 7. (**a**) The iris image whose upper part is occluded by eyelid; (**b**) the iris mask ROI and the six saliency points recovered by the proposed method; (**c**) a diagonal connection to connect each saliency points so that the line segments pass over the pupil region; (**d**) estimated pupillary boundary (shown in red color) recovered from the intersection of the line and inner part of the mask; (**e**) the pupillary boundary estimated by proposed method perfectly fits the pupil on iris ROI.

After that, all the points will be connected to other corresponding points so that several line segments, which go across the pupil can be formed. We perform a diagonal connection between two saliency points in order to make sure that the line passes through the pupil region, rather than goes horizontally. From those lines, we can get the saliency points for inner boundary by looking for the intersection between the diagonal lines and the pupil mask. To locate the circle of inner boundary accurately, we did the same thing as outer boundary estimation by recovering the pupillary circle parameter using Equation (2). The whole procedure is shown in Figure 8.

## 4. Experimental Results and Discussion

### 4.1. Database and Protocol

The database used in this experiment is CASIA-Iris-Thousands database [42]. It was established by the Institute of Automation, Chinese Academy of Science. It contains a total of 20,000 images from 1000 different subjects. The image resolution of this database is 640 × 480 pixels and each subject has the same number of the left eye and right eye images. All images in this database were captured with a close-up iris camera under near-infrared illumination (NIR). For training the model, we randomly split the data into training, validation, and testing with the ratio 80:10:10, respectively. The ground-truth masks of the CASIA-Iris-Thousands and UBIRIS.V2 are manually labeled using Labelme [43]. The proposed method was implemented in Matlab 2019b and for the iris mask estimation work, we follow the same training protocol as in Chen et al. [6]. The output of the IRUNet model has an image size 300 × 400. To equate the original images, we resized the iris mask to 640 × 480. Since the iris mask was not generated perfectly without any noises, we first converted the iris mask into binary images and did the morphological operation for the preprocessing step to eliminate the noises. We performed our experiments on a machine with Intel(R) Xeon(R) CPU E5–2620 and an NVIDIA 1080ti GPU with 11GB memory.

### 4.2. Performance Evaluation

To assess the performance of iris segmentation, we employ a common evaluation metric called intersection over union (IoU) [44,45]. The values of IoU are restricted to [0,1] interval, and 1 represents the perfect results (100% accuracy), while 0 is the worst results. The successful segmentation is defined when the value of IoU is higher than a predefined threshold. On the other hand, if the value of IoU is smaller than it, then the segmentation results are defined as inaccurate. In the experiment, we set the threshold to 0.8 to make the condition stricter. We compared the experimental results with some related works, which use the same database, including the conventional and CNN based methods to reflect the effectiveness of our algorithm, as shown in Table 1. In particular, Li et al. [4] proposed a new approach for doing iris segmentation by applying Faster RCNN to do the object detection and generates a bounding box followed by a Gaussian Mixture Model (GMM) to fit the potential inner region and then recovered five key points to locate the inner boundary. We also compared our results to the original U-Net [37] to measure the performance of proposed IR architecture.

A combination of enhanced MIGREP [8] and boundary point selection algorithm are also adopted to locate the outer boundary. MIGREP [8] proposed a new method to select boundary points for accurate localization on smart glasses device. This method designs a path that emitting rays radially outward from the inner center to locate the outer boundary. The starting points of emitting ray should be greater than the inner center and the endpoints should pass over the sclera region. Along with the emitting rays, the authors record the pixel intensity that has the maximal variation. Finally, the location corresponds to the edge of the outer boundary. A conventional method, the well-known circular Hough transform [46], is also used to compare with the performance of our algorithm. This method votes the circle candidates given the radius and then selects the local maxima value in the accumulator matrix. Circular Hough transform is often used to detect any curve shape in some imperfect images.

**Figure 9:**
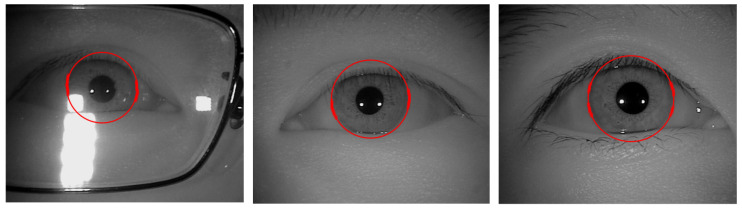
Example results of outer boundary estimation.

**Figure 10:**
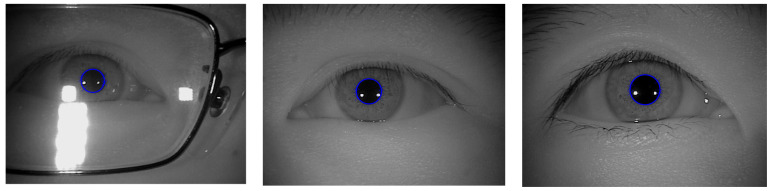
Example results of inner boundary estimation.

**Figure 11:**
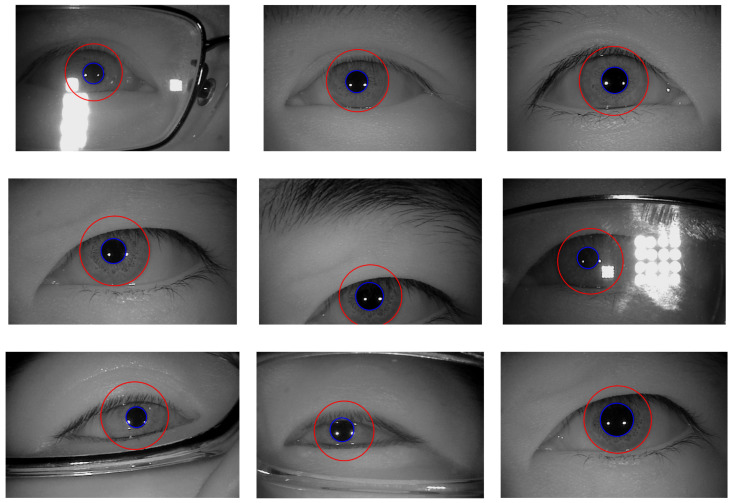
Example results of outer and inner boundary estimation.

**Figure 12:**
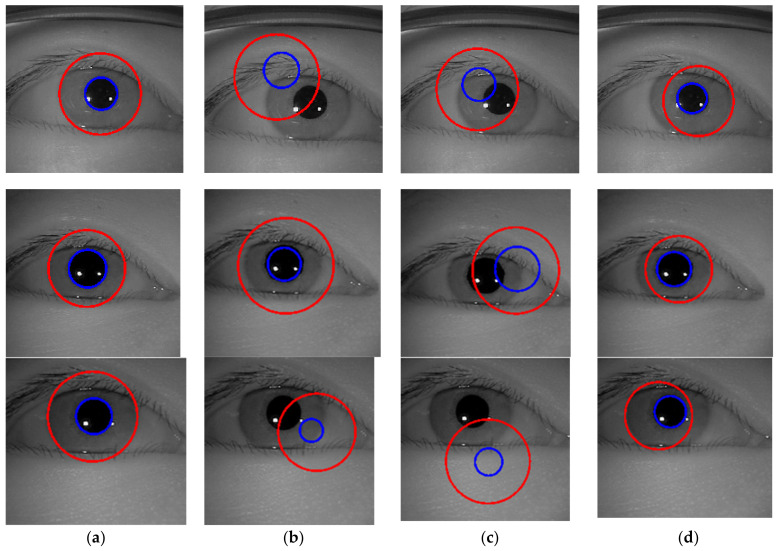
Outer and inner boundary estimation comparison of the proposed method with other works on challenging image. (**a**) Proposed method; (**b**) Li et al. [4]; (**c**) Li and Huang [8]; (**d**) Duda and Hart [46], respectively.

**Figure 13:**
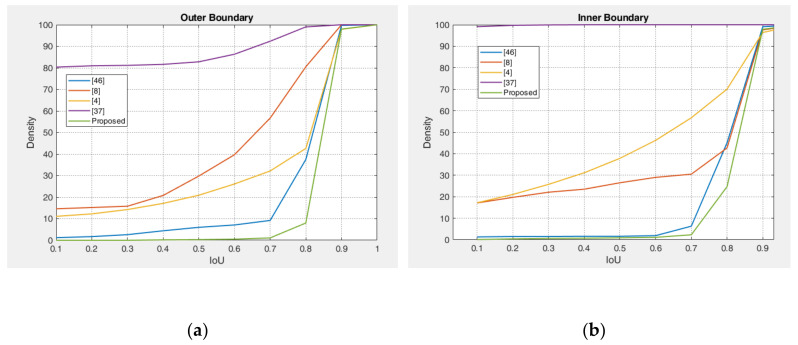
IoU cumulative distribution of proposed method on CASIA-Iris-Thousands database compare with other works. (**a**) Outer boundary distribution; (**b**) inner boundary distribution

The experimental results of the outer boundary, inner boundary estimation, and both boundary estimation plotted on original images are shown in Figure 9, Figure 10 and Figure 11, respectively. In order to evaluate the performance of the proposed method, we compared the experimental results in estimating the outer and inner boundary on very challenging images with other works as shown in Figure 12. Our proposed method outperforms the conventional and CNN based method by successfully recovering the accurate inner and outer boundary. The distribution of the mean IoU of the proposed method compared with other works is shown in Figure 13. However, during the experiments, due to the existence of images captured in non-cooperative environment, our proposed method cannot recover the inner and outer boundary accurately. The failed case can be caused by either the imperfect predicted mask from the output of the network, or because of the existence of very strong occlusion. It is very hard to locate enough number of saliency points in such cases, which might cause the segmentation to fail.

**Figure 14:**
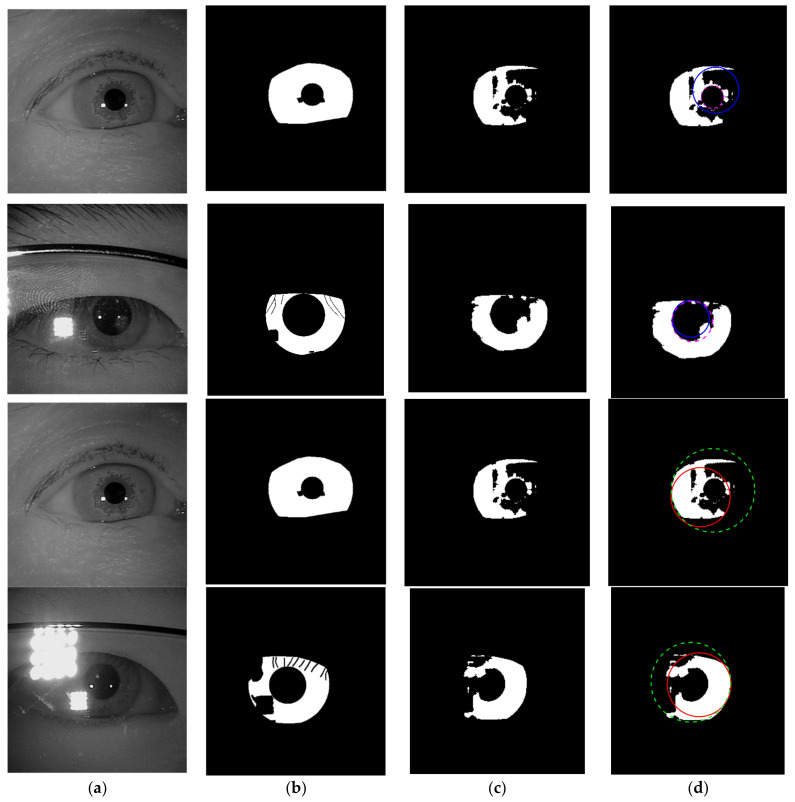
Failure cases because of the imperfect predicted masks. (**a**) Input images; (**b**) ground-truth masks; (**c**) predicted masks; (**d**) recover inner and outer boundary. The dashed lines denote the ground-truth and the solid lines denote the prediction. Row 1 and 2 show the result for inner boundaries and row 3 and 4 show the result for outer boundaries.

**Figure 15:**
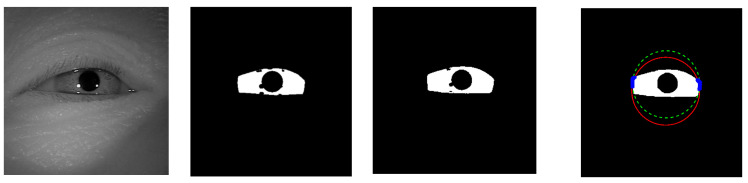

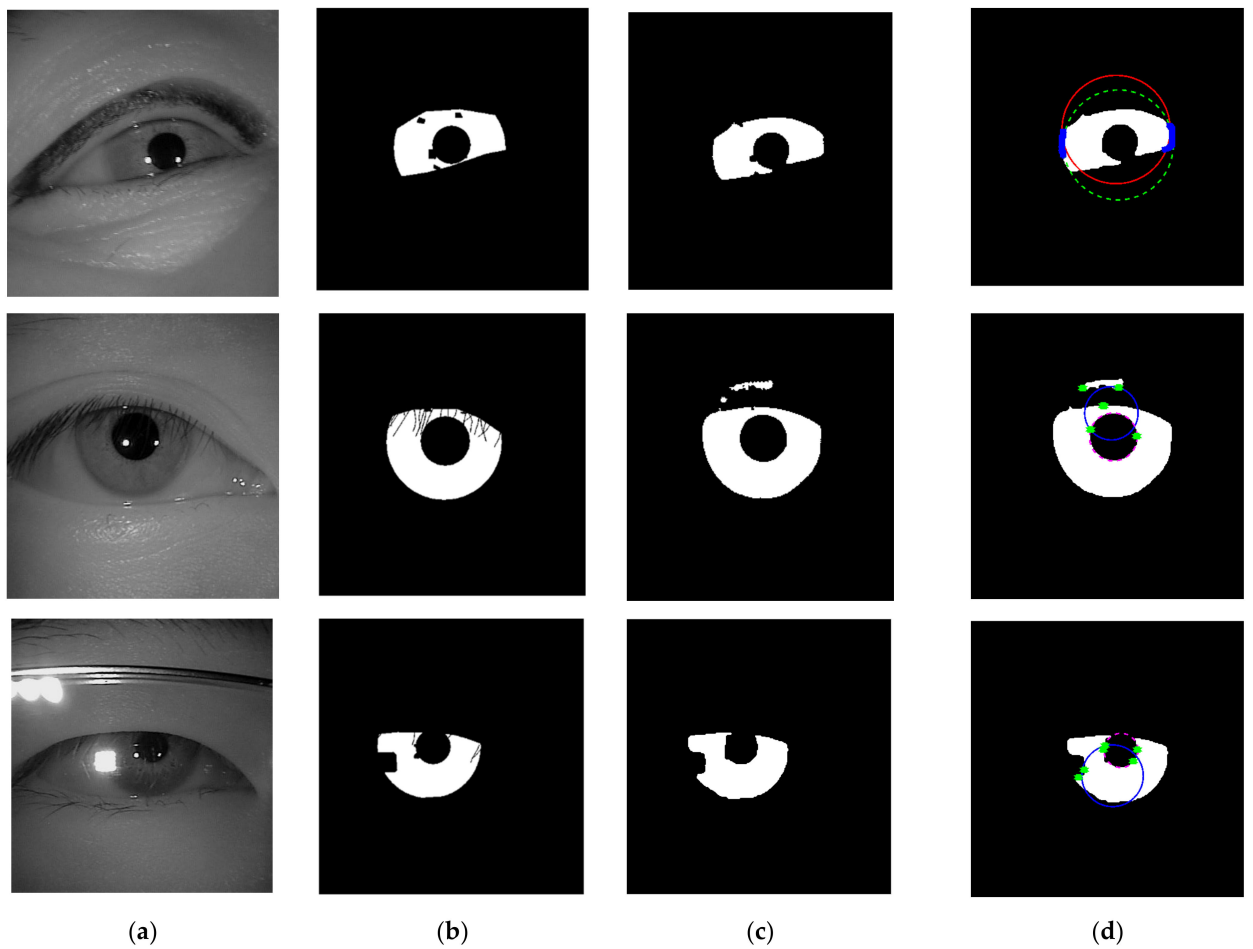
Failure cases because of the strong occlusion. (**a**) Input images; (**b**) ground-truth masks; (**c**) predicted masks; (**d**) estimated inner and outer boundary. The dashed lines denote the ground-truth and the solid lines denote the prediction. Blue dots denote the recovered saliency points of the iris, and green dots denote the recovered saliency points of the pupil.

**Figure 16:**
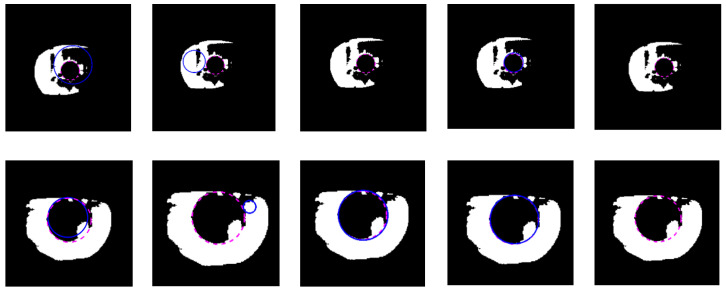

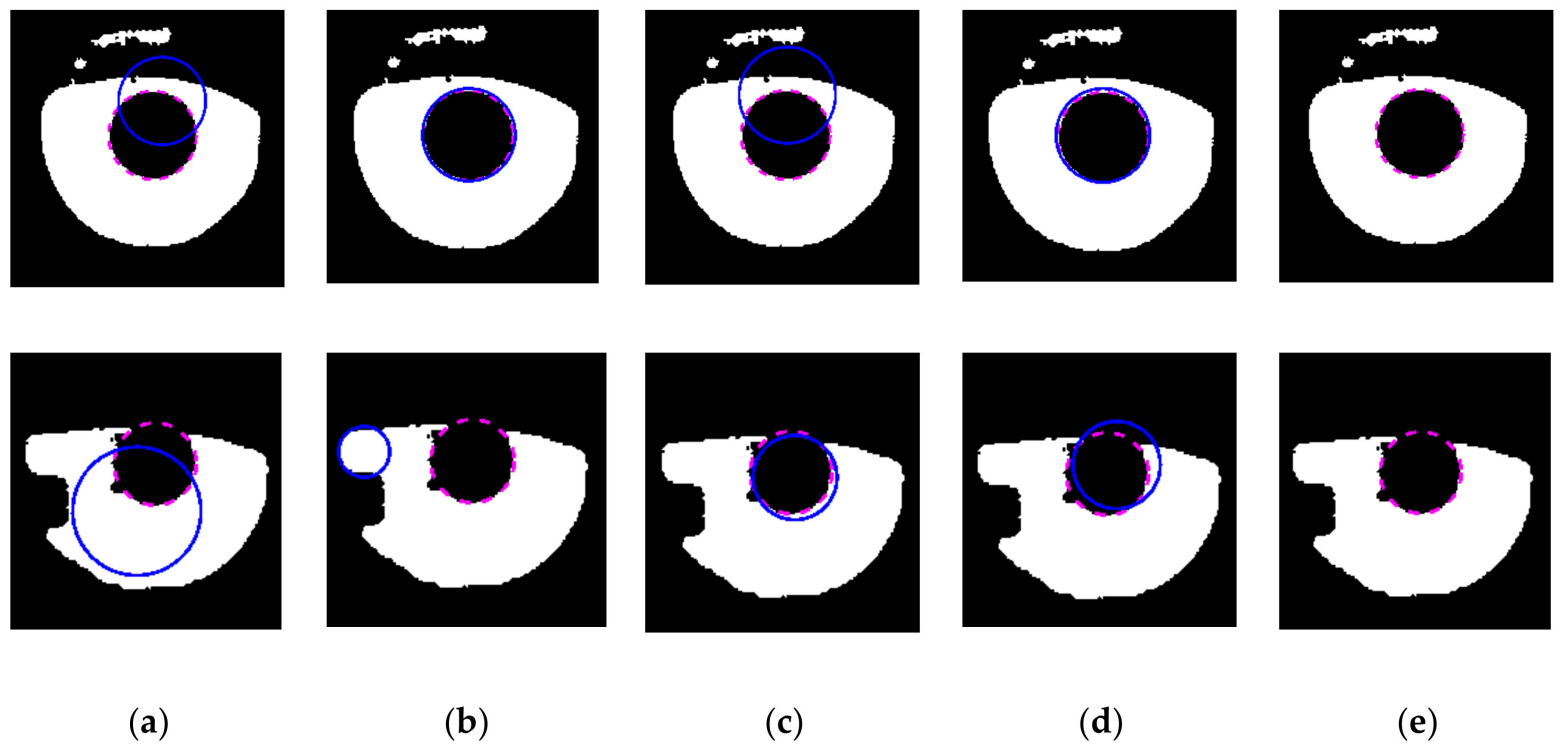
Failure cases on challenging images compare with other works to recover the inner boundary. (**a**) Proposed method; (**b**) Li et al. [4]; (**c**) Li and Huang [8]; (**d**) Ronneberger et al. [37]; (**e**) Duda and Hart [46]. The dashed line denotes the ground-truth and the blue line denotes the prediction of the inner boundary.

**Figure 17:**
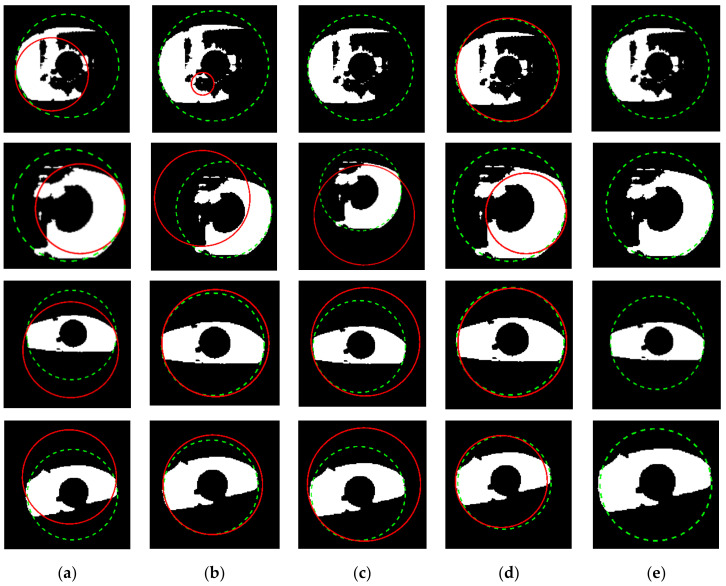
Failure cases on challenging images compare with other works to recover the outer boundary. (**a**) Proposed method; (**b**) Li et al. [4]; (**c**) Li and Huang [8]; (**d**) Ronneberger et al. [37]; (**e**) Duda and Hart [46]. The dashed line denotes the ground-truth, the red line denotes the prediction of the outer boundary.

**Figure 18:**
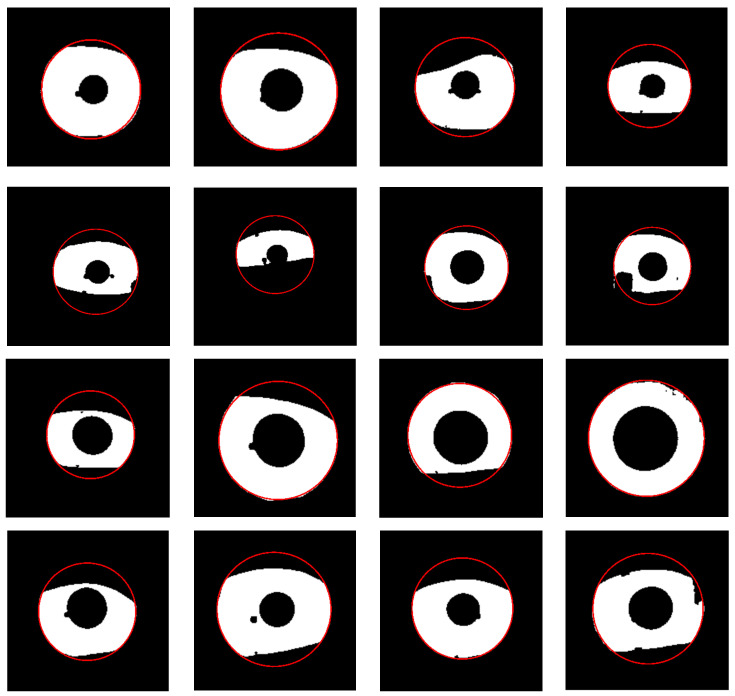
Visualization of predicted results on different iris shape.

**Figure 19:**
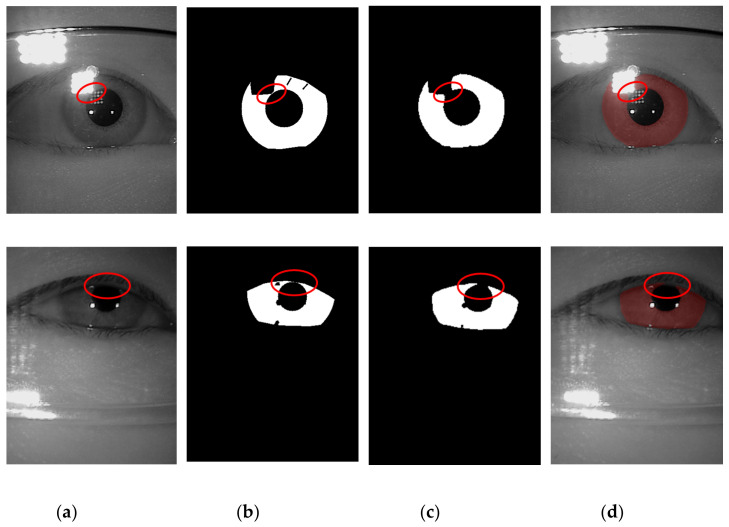
A pictorial example to show the robustness of the proposed method on the challenging images. (**a**) The input image; (**b**) the ground-truth masks; (**c**) the predicted masks; (**d**) the segmentation result plotted on the input images.

The example cases on challenging images caused by the incorrect iris masks are shown in Figure 14. Meanwhile, the failure cases due to the very strong occlusion are shown in Figure 15. The failure cases to recover the inner and outer boundary compare with other works are shown in Figure 16 and Figure 17, respectively. Nonetheless, Figure 18 shows the effectiveness of our proposed method to recover the boundary in various shapes of iris. The four columns of Figure 19 present an input iris image, the corresponding ground-truth mask, the corresponding prediction mask, and the plotted prediction mask on the input image. The red circle marks the non-iris area, which is labeled as iris pixels in the ground-truth mask, yet in the prediction masks, it is labeled as non-iris pixels. Thus, the example demonstrates the robustness of our proposed method since the generated mask fits closer to the true iris texture than the ground-truth mask.

## 5. Conclusions

Iris segmentation plays a crucial role in the whole process of iris recognition and directly affects the performance of the system. In this paper, we introduced a new algorithm to locate the outer and inner boundary of iris images accurately. By adopting the IRUNet model, we successfully generated an iris mask, which helps to estimate the boundary points more easily and more accurately. The experimental results suggested that the proposed iris segmentation algorithm outperforms other algorithms with the mean IoU value of outer boundary and inner boundary 98.9% and 97.7%, respectively. As for future work, we plan to explore the strategies to make the model more lightweight while keeping accuracy and efficiency as high as possible.

## Figures and Tables

**Table 1:** Comparison of segmentation accuracy with other works (intersection over union (IOU) threshold is defined as 0.8).

Methods	Outer Boundary	Inner Boundary	Execution Time Per Image
Circular Hough transform [46]	90.80%	93.70%	0.024
MIGREP [8]	43.3%	69.50%	0.011
Faster-RCNN [4]	67.84%	43.25%	0.05
U-Net [37]	7.6 %	0 %	0.007
Proposed method	98.9%	97.7%	0.007

## Data Availability

Publicly available datasets were analyzed in this study. This data can be found here: http://biometrics.idealtest.org/dbDetailForUser.do?id=4 (accessed on 17 February 2021).

## References

[1] Arsalan M., Naqvi R.A., Kim D.S., Nguyen P.H., Owais M., Park K.R. (2018). IrisDenseNet: Robust iris segmentation using densely connected fully convolutional networks in the images by visible light and near-infrared light camera sensors. Sensors.

[2] Arsalan M., Hong H.G., Naqvi R.A., Lee M.B., Kim M.C., Kim D.S., Kim C.S., Park K.R. (2017). Deep learning-based iris segmentation for iris recognition in visible light environment. Symmetry.

[3] Wang C., Muhammad J., Wang Y., He Z., Sun Z. (2020). Towards Complete and Accurate Iris Segmentation Using Deep Multi-Task Attention Network for Non-Cooperative Iris Recognition. IEEE Trans. Inf. Forensics Security.

[4] Li Y.-H., Huang P.-J., Juan Y. (2019). An efficient and robust iris segmentation algorithm using deep learning. Mob. Inf. Syst..

[5] Ma L., Zhang D., Li N., Cai Y., Zuo W., Wang K. (2012). Iris-based medical analysis by geometric deformation features. IEEE J. Biomed. Health Informat..

[6] Chen Y., Wang W., Zeng Z., Wang Y. (2019). An adaptive CNNs technology for robust iris segmentation. IEEE Access.

[7] Li Y.-H., Aslam M.S., Yang K.-L., Kao C.-A., Teng S.-Y. (2020). Classification of Body Constitution Based on TCM Philosophy and Deep Learning. Symmetry.

[8] Li Y.-H., Huang P.-J. (2017). An accurate and efficient user authentication mechanism on smart glasses based on iris recognition. Mob. Inf. Syst..

[9] Schnabel B., Behringer M. (2016). Biometric Protection for Mobile Devices is Now More Reliable: Research award for the development of an infrared LED for reliable iris recognition in smartphones and tablets. Optik Photonik.

[10] Woodard D.L., Pundlik S., Miller P., Jillela R., Ross A. On the fusion of periocular and iris biometrics in non-ideal imagery. Proceedings of the 20th International Conference on Pattern Recognition.

[11] Daugman J.G. (1993). High confidence visual recognition of persons by a test of statistical independence. IEEE Transact. Pattern Analysis Mach. Intell..

[12] Wildes R.P. (1997). Iris recognition: An emerging biometric technology. Proc. IEEE.

[13] Proença H., Alexandre L.A. (2006). Iris segmentation methodology for non-cooperative recognition. IEE Proc. Vis. Image Signal Process..

[14] Tan T., He Z., Sun Z. (2010). Efficient and robust segmentation of noisy iris images for non-cooperative iris recognition. Image Vis. Comput..

[15] Alvarez-Betancourt Y., Garcia-Silvente M. A fast iris location based on aggregating gradient approximation using QMA-OWA operator. Proceedings of the International Conference on Fuzzy Systems.

[16] Peláez J.I., Doña J.M. (2006). A majority model in group decision making using QMA–OWA operators. Int. J. Intell. Syst..

[17] Ghodrati H., Dehghani M.J., Helfroush M.S., Kazemi K. Localization of noncircular iris boundaries using morphology and arched Hough transform. Proceedings of the 2nd International Conference on Image Processing Theory, Tools and Applications.

[18] Canny J. (1986). A computational approach to edge detection. IEEE Trans. Pattern Anal. Mach. Intell..

[19] Wang X.-c., Xiao X.-m. An Iris segmentation method based on difference operator of radial directions. Proceedings of the 2010 Sixth International Conference on Natural Computation.

[20] Jin L., Xiao F., Haopeng W. Iris image segmentation based on K-means cluster. Proceedings of the IEEE International Conference on Intelligent Computing and Intelligent Systems.

[21] Yan F., Tian Y., Wu H., Zhou Y., Cao L., Zhou C. Iris segmentation using watershed and region merging. Proceedings of the 9th IEEE Conference on Industrial Electronics and Applications.

[22] Roerdink J.B., Meijster A. (2000). The watershed transform: Definitions, algorithms and parallelization strategies. Fundamenta Informaticae.

[23] Abate A.F., Frucci M., Galdi C., Riccio D. (2015). BIRD: Watershed based iris detection for mobile devices. Pattern Recognit. Letters.

[24] Radman A., Zainal N., Suandi S.A. (2017). Automated segmentation of iris images acquired in an unconstrained environment using HOG-SVM and GrowCut. Digital Signal Process..

[25] Banerjee S., Mery D. Iris segmentation using geodesic active contours and grabcut. Proceedings of the Image and Video Technology.

[26] Rongnian T., Shaojie W. Improving iris segmentation performance via borders recognition. Proceedings of the 2011 Fourth International Conference on Intelligent Computation Technology and Automation.

[27] Liu N., Li H., Zhang M., Liu J., Sun Z., Tan T. Accurate iris segmentation in non-cooperative environments using fully convolutional networks. Proceedings of the 2016 International Conference on Biometrics (ICB).

[28] Hofbauer H., Jalilian E., Uhl A. (2019). Exploiting superior CNN-based iris segmentation for better recognition accuracy. Pattern Recognit. Lett..

[29] Kerrigan D., Trokielewicz M., Czajka A., Bowyer K.W. Iris recognition with image segmentation employing retrained off-the-shelf deep neural networks. Proceedings of the 2019 International Conference on Biometrics (ICB).

[30] Jalilian E., Uhl A. (2017). Iris segmentation using fully convolutional encoder–decoder networks. Deep Learning for Biometrics.

[31] Lian S., Luo Z., Zhong Z., Lin X., Su S., Li S. (2018). Attention guided U-Net for accurate iris segmentation. J. Vis. Commun. Image Represent..

[32] Bazrafkan S., Thavalengal S., Corcoran P. (2018). An end to end deep neural network for iris segmentation in unconstrained scenarios. Neural Netw..

[33] Arsalan M., Kim D.S., Lee M.B., Owais M., Park K.R. (2019). FRED-Net: Fully residual encoder–decoder network for accurate iris segmentation. Expert Syst. Appl..

[34] Lozej J., Meden B., Struc V., Peer P. End-to-end iris segmentation using u-net. Proceedings of the 2018 IEEE International Work Conference on Bioinspired Intelligence (IWOBI).

[35] Wu X., Zhao L. (2019). Study on iris segmentation algorithm based on dense U-Net. IEEE Access.

[36] Zhang W., Lu X., Gu Y., Liu Y., Meng X., Li J. (2019). A robust iris segmentation scheme based on improved U-net. IEEE Access.

[37] Ronneberger O., Fischer P., Brox T. U-net: Convolutional networks for biomedical image segmentation. Proceedings of the International Conference on Medical Image Computing and Computer-assisted Intervention.

[38] Gautam G., Mukhopadhyay S. (2020). Challenges, taxonomy and techniques of iris localization: A survey. Digit. Signal Process..

[39] Wang C., He Y., Liu Y., He Z., He R., Sun Z. Sclerasegnet: An improved u-net model with attention for accurate sclera segmentation. Proceedings of the 2019 International Conference on Biometrics (ICB).

[40] Chen L.-C., Papandreou G., Schroff F., Adam H. (2017). Rethinking atrous convolution for semantic image segmentation. arXiv.

[41] MacQueen J. Some methods for classification and analysis of multivariate observations. Proceedings of the fifth Berkeley symposium on mathematical statistics and probability.

[42] CASIA Iris Image Database. http://biometrics.idealtest.org/dbDetailForUser.do?id=4.

[43] Kentaro Wada K. LabelMe: Image polygonal annotation with Python. https://github.com/wkentaro/labelme.

[44] Chen L.-C., Zhu Y., Papandreou G., Schroff F., Adam H. Encoder-decoder with atrous separable convolution for semantic image segmentation. Proceedings of the European conference on computer vision (ECCV).

[45] He K., Gkioxari G., Dollár P., Girshick R. Mask r-cnn. Proceedings of the IEEE international conference on computer vision (ICCV).

[46] Duda R.O., Hart P.E. (1972). Use of the Hough transformation to detect lines and curves in pictures. Commun. ACM.

